# Point Me in the Right Direction: Same and Cross Category Visual Aftereffects to Directional Cues

**DOI:** 10.1371/journal.pone.0141411

**Published:** 2015-10-28

**Authors:** Sarah Maeve Cooney, Alanna O’Shea, Nuala Brady

**Affiliations:** 1 School of Psychology, University College Dublin, Belfield, Dublin, Ireland; 2 School of Biomolecular and Biomedical Science, University College Dublin, Belfield, Dublin, Ireland; University of Udine, ITALY

## Abstract

Of the many hand gestures that we use in communication pointing is one of the most common and powerful in its role as a visual referent that directs joint attention. While numerous studies have examined the developmental trajectory of pointing production and comprehension, very little consideration has been given to adult visual perception of hand pointing gestures. Across two studies, we use a visual adaptation paradigm to explore the mechanisms underlying the perception of proto-declarative hand pointing. Twenty eight participants judged whether 3D modeled hands pointed, in depth, at or to the left or right of a target (test angles of 0°, 0.75° and 1.5° left and right) before and after adapting to either hands or arrows which pointed 10° to the right or left of the target. After adaptation, the perception of the pointing direction of the test hands shifted with respect to the adapted direction, revealing separate mechanisms for coding right and leftward pointing directions. While there were subtle yet significant differences in the strength of adaptation to hands and arrows, both cues gave rise to a similar pattern of aftereffects. The considerable cross category adaptation found when arrows were used as adapting stimuli and the asymmetry in aftereffects to left and right hands suggests that the adaptation aftereffects are likely driven by simple orientation cues, inherent in the morphological structure of the hand, and not dependent on the biological status of the hand pointing cue. This finding provides evidence in support of a common neural mechanism that processes these directional social cues, a mechanism that may be blind to the biological status of the stimulus category.

## Introduction

With relative ease and little reflection we readily follow numerous cues that direct our focus of attention. These directional cues can be biological such as hand gestures, head orientation and eye gaze or symbolic non-biological cues, like arrows. Of the various biological cues to social attention that people use, hand pointing is a meaningful social gesture that can convey a variety of communicative intentions [1]. Comprehension of pointing gestures provides an integral foundation for developing social reciprocity. The intention of the gesture is often aligned with the type of communicative point: imperative pointing is used to request an object whereas declarative pointing is used to direct another’s attention upon an object or event in the environment [1,2].

Producing declarative pointing gestures with the index finger begins to emerge toward the end of the first year of infancy [1,3–5], is predominantly right handed [6] and is predictive of positive vocabulary acquisition [7,8]. Infants as young as 4.5 months have been shown to be sensitive to pointing direction towards a distal referent [9]. A developmental delay in producing and comprehending pointing gestures is often used as a diagnostic marker for Autism Spectrum Disorder (ASD) [10]. Pointing, like eye gaze, is a deictic gesture that permits the signaler to indicate to a recipient an object or target of interest. The resulting convergence of eye gaze between the two interacting parties facilitates a mutual understanding of a common focus of interest, understood as joint attention [11,12].

Numerous studies have examined the developmental trajectory of pointing production and comprehension. By comparison, very little consideration has been given to adult perception of hand pointing gestures. While interacting with others, our visual system is constantly processing a number of biological directional cues that serve to establish and maintain joint attention [13]. Converging evidence suggests that some of these biological cues, such as eye gaze direction [14], body orientation [15] and head orientation [16], are represented at a relatively high-level of visual processing. This paper asks if the visual system selectively codes for hand pointing direction. If so, is this done at a high level of visual processing? To determine if a selective population of neurons represents hand-pointing direction in the brain we employ visual adaptation. Visual adaptation provides a well-established method [15,17–20] to study how the visual system codes for these directional cues.

When repeatedly presented with a stimulus, adaptation aftereffects manifest as perceptual biases that result from reduced neuronal sensitivity to specific features of that stimulus [20]. For example, adaptation to a leftward tilted line changes our perception of the orientation of a subsequently presented vertical line so that it appears to tilt in the opposite direction. The tilt aftereffect [21] and visual adaptation aftereffects in general reflect the continuous adjustment or recalibration of the response properties of neurons tuned to specific stimulus features in the environment [18,20]. Perceptual aftereffects have been found for basic stimulus features such as motion [22] and orientation [21] and to higher level qualities such as face identity [23], face viewpoint [16,19] and gender [24,25]. For instance, after participants adapted to a side view of a human face their judgments of forward facing test stimuli shifted in the opposite direction [16]. Fang and He [19] found that this face viewpoint aftereffect did not transfer between object groups, such that adapting to a face did not produce a perceptual shift in judging the viewpoint of a selection of non-biological control stimuli such as cars and abstract wireframe objects. However, cross category aftereffects have been reported for the representation of gender, such that adapting to a male face results in a perceptual shift in the perception of body gender away from the gender of the adaptor and vice versa [25]. Interpretation of this cross category aftereffect as evidence of neuronal populations tuned to the higher order concept of gender are supported by further research [24] where prolonged viewing of gender-specific non biological objects (e.g., lipstick, shoes, items of clothing etc.), induced a shift in the perception of face gender. There is considerable potential of cross category adaptation aftereffects to examine neural sensitivity to high-level stimulus features. Here, we use the technique to examine visual coding of hand pointing direction, a biological directional cue, and ask whether this type of directional cue is visually coded by a separate neural mechanism than arrows, a non-biological directional cue.

The limited amount of research on pointing comprehension in adults has almost exclusively employed attention modulation paradigms [26–30] such as the Posner visual cueing task (1980), Stroop type interference paradigms and eye movement tracking, in order to examine how pointing along with other social cues can modulate attention. With particular consideration given to the biological versus non-biological features of the social cues, a number of studies have reported that hand pointing direction, like eye gaze direction [27] exerts automatic control on the observers visual attention [30–32]. It is not only biological hand pointing and eye gaze cues that have been shown to modulate visual attention; social symbols such as arrows and directional words have been found to produce comparable attentional effects [33,34].These studies provide evidence of a common neural mechanism that processes these social cues that may be blind to the biological status of the stimulus category.

In contrast, studies that have employed more complex attention-orienting paradigms that require a heavier cognitive load, to examine the influence of biological versus non-biological cues in capturing visual attention have provided evidence to support separate attentional processing of eye gaze direction, hand pointing direction, and arrows [29,35]. One study [35] combined visual adaptation with attention orienting to examine the influence gaze perception and hand-pointing perception has on attentional shifts in the direction of observed gaze. They found that repeated exposure to averted gaze but not to hand pointing direction resulted in subsequent weakened gaze cueing in the adapted direction while gaze cueing in the unadapted direction remained at normal attention cueing levels. This suggests that eye gaze and hand pointing direction are coded separately as cues to social attention.

Here we employ an adaptation paradigm rather than an attentional cueing paradigm to explore whether two cues to social attention, index finger pointing gesture and a symbolic arrow, are coded separately in the visual system or in common. Both same-category and cross-category adaptation are used to examine, for the first time, the visual representation of hand pointing direction. We ask two main questions, does adapting to hand pointing direction like other biological cues to joint attention such as eye gaze, head orientation and body orientation elicit a perceptual aftereffect? And, if so, are the aftereffects indicative of separate groups of neurons that code for this particular directional body cue or does the directional information alone elicit a perceptual shift? If hand-pointing direction is represented in high level vision then cross adapting to a non-biological directional cue such as arrows should not elicit a shift in the perception of hand pointing direction.

## Materials and Methods

### Participants

Twenty-eight (14 female) right-handed volunteers from the UCD student population (mean age of years, 25.54, SD = 6.30 years) received fifteen euros for their participation. All were naïve to the purpose of the experiments and had normal or corrected to normal vision. The study was approved by the University College Dublin, Human Research Ethics Committee, Humanities (HREC); in accordance with the Declaration of Helsinki all participants gave written, informed consent and were advised of their right to withdraw from the study at any time without prejudice.

### Stimuli

The stimuli were computer-generated images of left and right hands with a portion of the forearm visible, positioned in a pointing gesture, with the index finger pointing in depth at an object, a kitchen skillet. All stimuli were created using 3D animation software Poser ^®^ http://poser.smithmicro.com.

**Fig 1:**
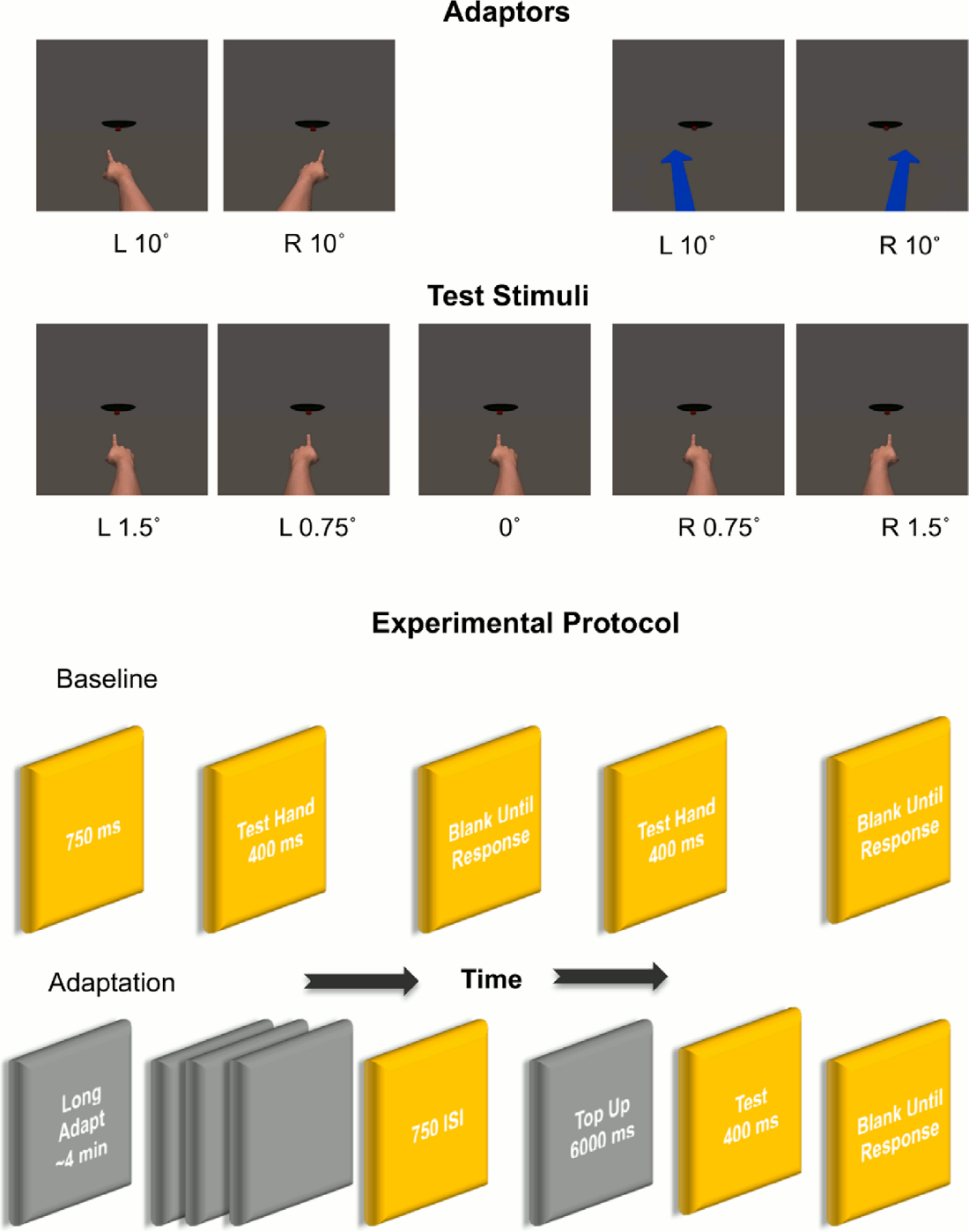
*Top Panel*: Examples of test stimuli (pointed hands), and adaptors, (arrow and pointed hands) at different orientations. ***Bottom Panel***: Sample of the experimental protocol. This included a baseline phase where participants judged the pointing direction of test hands at 5 orientations 0°, 0.75° right, 0.75° left, 1.5° right, 1.5° left. Followed by a long adaptation phase where participants viewed a series of adaptors (either hands or arrows). The long adaptation period, ~ 4mins, was immediately followed by a post-adapt top up and test block. Each top up adaptor—left and right hands (*same-adaptation)* or arrows (*cross-adaptation*) pointing 10° left or right of the skillet—was presented for 6000ms followed by a test stimulus for 400ms.

In both the *same-category* and *cross-category* variants of the adaptation experiment the test stimuli depicted 10 hands in total (5 right and 5 left hands) pointing directly at the handle of the skillet (0°) and 0.75° and 1.5° to the left or right of the skillet. These angles were chosen after a pilot study to gauge participants’ ability to discriminate pointing direction in this task. In the *same-category* adaptation variant, the adapting stimuli were 1 left and 1 right hand pointing in depth 10° to the left or right of the skillet. In the *cross-category* adaptation variant, the adaptor was changed to an arrow pointing in depth 10° to the left or 10° to the right of the skillet (See Fig 1).

All images were rendered in colour at 978 x 978 pixels and subtended, vertically, ~27° of visual angle at a viewing distance of ~60cm. The distance from the tip of the finger (when in the 0° position) to the skillet handle was ~5.2° of visual angle. The stimuli were presented and participants’ responses recorded using Presentation^®^ (http://neurobs.com) running on a Dell XPS-8300 PC with a screen size of 19 inches and a display resolution of 2048 by 1152 at 60 Hz.

### Procedure

In both the *same category* and *cross category* adaptation variants of the experiment participants completed 2 experimental phases, a baseline phase and an adaptation phase, which were repeated for the 10° left and 10° right adaptors. To ensure no carry over adaptation effects participants had a 10-minute break between the right and left adaptation phases, and they completed the *same category* and the *cross category* adaptation variants of the experiment at least 1 day apart. Running order was also counterbalanced so that half the participants completed the same category variant of the experiment first and half completed the cross category first.

In each of the two baseline phases (one each before adaptation to right and to left pointing adaptors) all 10 hands (a right and left hand, each shown at 1.5° and 0.75° left, 0°, 0.75° and 1.5° right) were repeated 5 times for 50 trials, so that the combined number of baseline trials was 100. Baseline trials began with a central fixation cross for 750ms followed by a test hand for 400ms. The screen was then blanked until the participant responded using the number pad keys 1, 2 and 3 to indicate whether they perceived the hand as pointing to the left of, directly at or to the right of the skillet handle respectively. Presentation order was pseudo-randomized.

The adaptation phase started with an adaptation period of ~ 4mins during which the adaptation stimuli (a left and a right hand pointing 10° to the right or left of the skillet handle) were presented 25 times each for 4000ms each followed by a blank 750ms ISI. Adaptation direction was counterbalanced so that half the participants adapted to leftward pointing stimuli first and half to rightward pointing stimuli first. The adaptation period was immediately followed by a post-adapt top up and test block of 50 trials. Each top up adaptor—left and right hands (*same-adaptation)* or arrows (*cross-adaptation*) pointing 10° left or right of the skillet—was presented for 6000ms followed by a test stimulus for 400ms. The word ‘RESPOND’ was printed beneath each test stimulus and participants judged whether the test hands were pointing to the left of, directly at or to the right of the skillet using the keys on the number pad. The order of the 50 test trials was pseudo-randomized.

## Results

**Fig 2:**
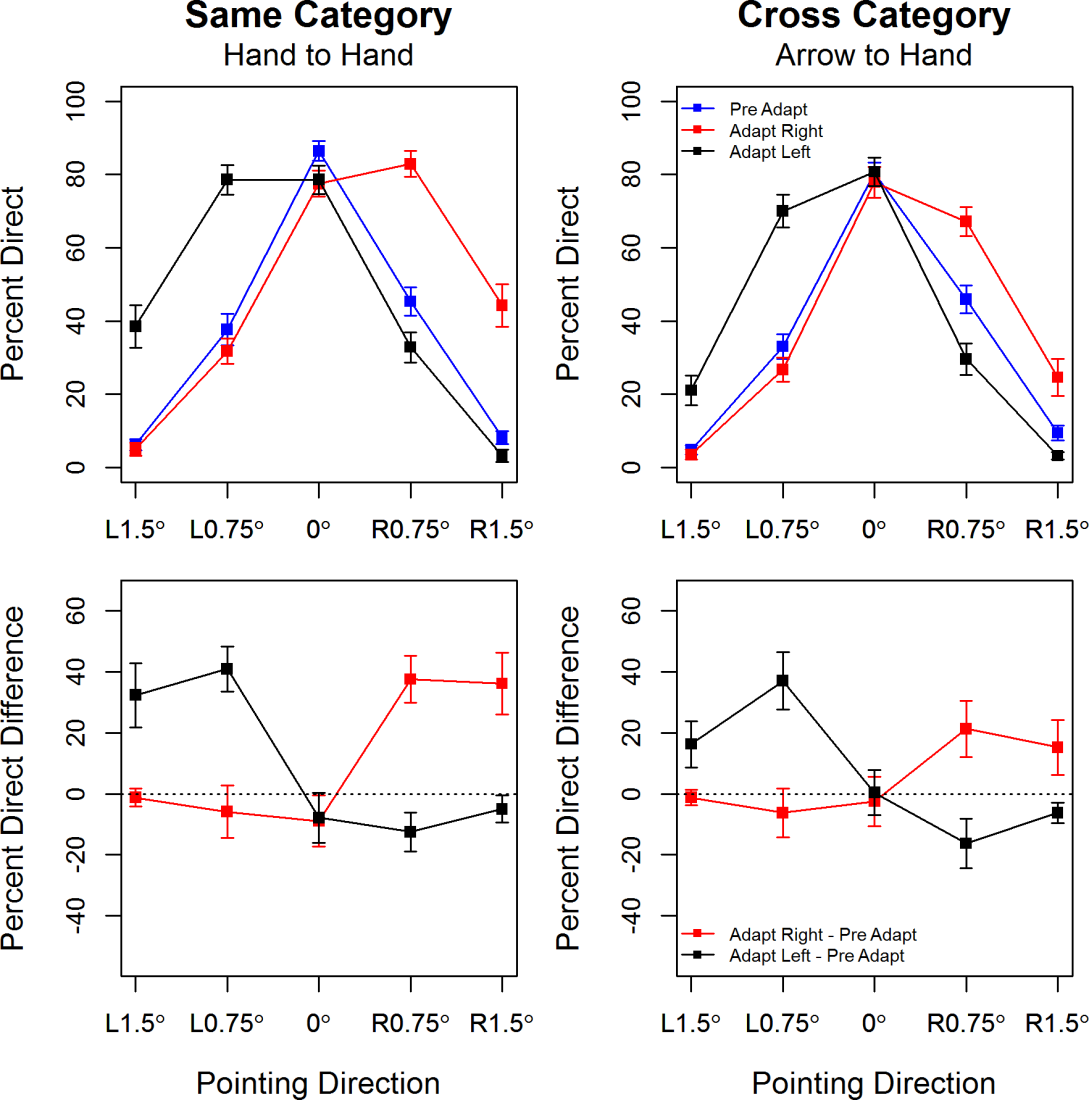
*Top Panel*: Percentage direct responses plotted by hand pointing direction with separate traces for pre-adapt (blue), post-adapt right (red) and post-adapt left (black). Error bars show +/1 S.E.M. ***Bottom Panel***: Mean difference in the percentage of direct responses pre- and post-adaptation by pointing direction, with separate traces for adaptation to rightward (red) and leftward (black) pointing adaptors. Error bars show 95% confidence intervals around the point estimates, the dotted black line marks an effect size of zero. Same and cross category variants of the experiment are shown in the left and right panel respectively.

The percentage of ‘direct’ responses was analysed in R [36] using ANOVA with within-subjects factors of test hand *Pointing Direction* (5 levels), *Adaptation Condition* (pre-adapt, post-adapt right, post-adapt left), and *Adaptation Type* (same-category, cross-category)) and a between-subjects factor of *Order of Adaptation* (adapt to hands first, adapt to arrows first). Greenhouse-Geisser corrections were used when Mauchlys Test for Sphericity was significant and effect sizes are given by generalized eta squared (*η* ^*2*^_*G*_) [37]. Following Cumming [38], significant interactions are explored by plotting and reporting point estimates and associated confidence intervals (Fig 2). In addition, significant interactions were further analysed using planned comparisons with Bonferroni-correction.

In the omnibus test the main effect of *Order of Adaptation* was not significant, F(1,26) = 3.55, *p* = 0.07, *η2G* = 0.03, and none of the higher order effects involving *Order of Adaptation* were significant, e.g., for the 4-way interaction of *Order of Adaptation* * Direction * Condition * Type, *p* = 0.80. Therefore the data are collapsed across the two groups of participants (those who adapted to hands first and those who adapted to arrows first) in Fig 2 below

The percentage of trials on which participants perceived the test hands as pointing directly at the skillet handle is plotted by hand pointing direction in the top panel of Fig 2. There is evidence of strong adaptation in the *same-category* condition. At baseline participants show high accuracy in judging the direction of hands which are pointing directly at the skillet and 1.5° to its left or right, but often judge hands pointing 0.75° to the left or to the right as pointing directly at the target. After adapting to hands pointing 10° to the right or to the left of the target, the response curves shift in a way that is indicative of negative aftereffects as outlined by Webster and McLeod [39]. First, hands that are pointing in the same direction as the adapting stimulus are now more likely to be perceived as pointing directly at the target, and this is particularly marked for the smallest pointing angle (0.75°). Secondly, and in contrast, hands that are pointing in the opposite direction to the adapting stimulus are now more likely to be correctly perceived as pointing in that direction. These characteristics of adaptation are also evident in the lower panel of Fig 2 in which we plot point estimates of effect sizes (the mean difference in the percentage of straight ahead responses pre- and post-adaptation) with associated 95% CIs. The effects are positive for test hands that point in the same direction as the adaptors and negative for test hands that point in the opposite direction to the adaptors.

There is similar evidence of strong adaptation in the *cross-category* condition where arrows pointing to the right or left of the skillet handle serve as the adapting stimuli. Test hands that are pointing in the same direction as the adapting arrows are now more likely to be perceived as pointing directly at the target. And accuracy is sharpened a little for test hands pointing in the opposite direction to the adapting arrows, i.e., after right (*left*) adaptation participants are more likely to correctly perceive 0.75° left (*right)* pointing hands as pointing to the left (*right*).

Statistical analyses confirm these observations. The omnibus test showed that the 3-way interaction of Pointing Direction*Adaptation Condition*Adaptation Type, F(8,208) = 5.39, *p* < 0.001, *η2G* = 0.03, was significant. This suggests that the strength and pattern of adaptation varied with the type of adaptation, hand-to-hand or arrow-to-hand. Further analyses were performed on the *same-category* and *cross-category* data sets separately.

ANOVA for the *same-category* data showed significant main effects of *Pointing Direction*, F(4,108) = 134.93, *p* ~ 0, *η2G* = 0.61, and of *Adaptation Condition*, F(2,54) = 27.50, *p* ~ 0.0, *η2G* = 0.06, whose interpretation is qualified by a significant Pointing Direction* Adaptation Condition interaction, F(8,216) = 53.35, *p* ~ 0, *η2G* = 0.42. Planned comparisons showed a significant change in the percentage of ‘pointing directly at’ responses at 0.75° right, t(27) = 6.38, *p* ~ 0.0, ES = 37.5% [29.88, 45.12] and at 1.5° right t(27) = 7.28, *p* ~ 0.0, ES = 36.07% [25.90, 46.24], between baseline and adaptation to rightward pointing hands. Planned comparisons between baseline and adaptation to leftward pointing hands showed a significant change in the percentage of ‘pointing directly at’ responses at 1.5° left, t(27) = 6.32, *p* ~ 0.0, ES = 32.32% [21.83, 42.82], at 0.75° left, t(27) = 11.33, *p* ~ 0.0, ES = 40.89% [33.49, 48.30] and at 0.75° right, t(27) = -4.07, *p* = 0.0004, ES = -12.5% [-18.79: -6.20].

ANOVA for the *cross-category* data likewise showed significant main effects of *Pointing Direction* F(4,108) = 186.06, *p* ~ 0, *η2G* = 0.67 and of *Adaptation Condition*, F(2,54) = 11.69, *p* ~ 0.0, *η2G* = 0.02 whose interpretation is qualified by a significant Pointing Direction* Adaptation Condition interaction, F(8,216) = 30.94, *p* ~ 0, *η2G* = 0.30. Planned comparisons showed a significant change in the percentage of ‘pointing directly at’ responses at 0.75° right, t(27) = 4.78, *p* = 0.00005, ES = 21.25% [12.13, 30.37], and at 1.5° right, t(27) = 3.47, *p* = 0.0017, ES = 15.17% [6.22, 24.14], between baseline and adaptation to rightward pointing arrows. Planned comparisons between baseline and adaptation to leftward pointing arrows showed a significant change in the percentage of ‘pointing directly at’ responses at 1.5° left, t(27) = 4.39, *p* = 0.0002, ES = 16.25% [8.65, 23.85], at 0.75° left, t(27) = 8.09, *p* ~ 0.0, ES = 36.96% [27.59, 46.33] and at both 0.75° right, t(27) = -4.13, *p* = 0.0003, ES = -16.25% [-24.32: -8.18], and 1.5° right, t(27) = -3.77, *p* = 0.0008, ES = -6.25% [-9.65: -2.85]. A comparison of these planned comparisons results across the same-category and cross-category adaptation conditions, along with inspection of the lower panels of Fig 2, reveals the rather subtle effect of the Adaptation Type*Adaptation Condition*Pointing Direction interaction, i.e., same-category (hand-hand) adaptation generally leads to slightly higher changes in the percentage of ‘pointing straight at’ responses than does cross-category (arrow-hand) adaptation

**Fig 3:**
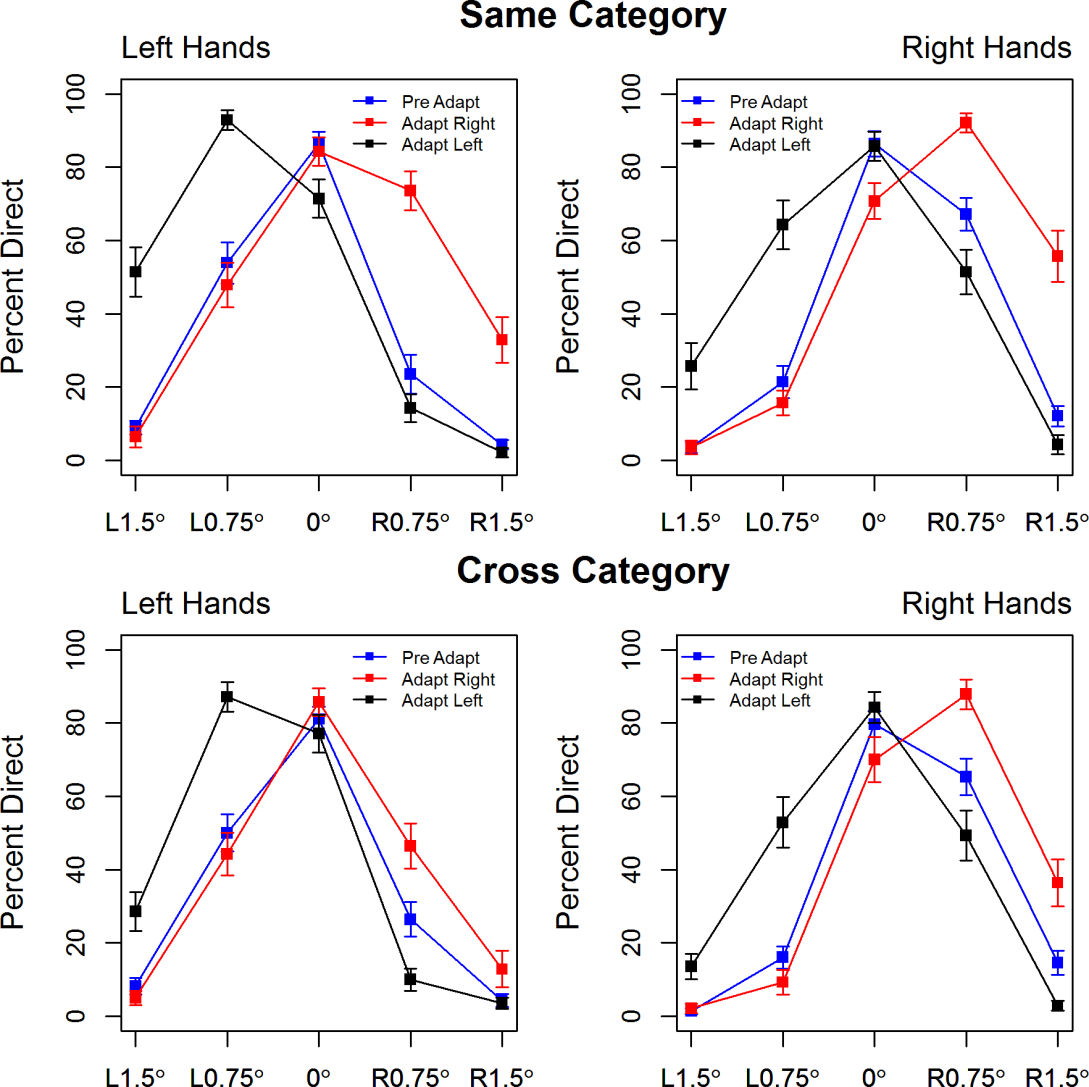
Percentage direct responses are re-plotted by hand pointing direction in the hand-to-hand (top panel) and arrow-to-hand (bottom panel) tasks, separately for left and right test hands. These plots illustrate an asymmetry in baseline (blue) performance such that participants are more accurate in judging the pointing direction of leftward pointing right hands and rightward pointing left hands when the task is most difficult. Error bars show +/1 S.E.M.

Fig 3 re-plots the percentage of direct responses by hand pointing direction separately for left and right test hands to explore the role of hand shape in participants’ judgments of hand pointing direction. At baseline, and when the task is most difficult (0.75°), participants are more accurate in judging the direction of leftward pointing right hands than of rightward pointing right hands and, similarly, they are more accurate in judging the direction of rightward pointing left hands than of leftward pointing left hands. This perceptual bias is also seen in the post-adaptation profiles where the shift in the ‘neutral point’ (the shift in which hand is most often perceived as pointing directly at the target) is hand specific, with a leftward shift for left hands and a rightward shift for right hands. This suggest that task performance is not based on a simple vernier acuity cue but that participants take both hand shape and index finger orientation into account in judging where the hand is pointing relative to the object.

## Discussion

This study presents, for the first time, evidence of rapid visual adaptation to hand pointing direction. Adapting to hands pointing away from an object resulted in a shift in the perception of the direction of subsequently presented hand pointing stimuli away from the direction of the adaptor, a key characteristic of negative aftereffects [39].

The novel direction specific high level adaptation aftereffect reported here has, to date, been observed for three other types of visual social cues; eye gaze direction[14], head orientation [16,19] and body orientation [15]. In concluding that these aftereffects reflect the operation of relatively high-level visual processing, researchers typically adjust either the size [15] or the orientation [40] of the adapting stimuli relative to the test stimuli so as to rule out low-level, retinotopic effects. Here the adapting stimuli (hands or arrows pointing 10° to the right or left of the target) differ in orientation to and are non-overlapping with the test stimuli (hands pointing 0° or 0.75° and 1.5° to the right or left of the target). As such, the aftereffects we report are unlikely to reflect retinotopic adaptation. Instead, this strong aftereffect provides initial evidence for the existence of neurons in the visual system tuned to hand pointing direction.

In response to a reviewer, we note that the pattern of responses before and after adaptation cannot be explained by a stimulus response compatibility effect, such as the Simon effect. According to the Simon effect, responses should be more accurate when participants press a response key that is located on the same side as the stimulus. In this study participants used the number pad which is located to the right of the keyboard, yet the same pattern of accuracy is seen for rightward and leftward pointing hands. In fact, examination of Fig 3 shows that participants are more accurate at judging the direction of right hands pointing leftward and left hands pointing rightward and find the task more difficult for right hands pointing rightward and left hands pointing leftward. This suggests that the overall shape of the hand is important to the task.

Given that the directional information present in the adapting stimulus is paramount to this aftereffect, we asked, whether it is the specific configuration of the pointed hand that is responsible or whether adaptation to a non-biological cue to direction elicits a comparable perceptual shift in the perception of hand pointing. The results show that when adapting to an arrow oriented away from the object, participants subsequent perception of hands pointing in the same direction were also repelled away from the direction of the arrow adaptor. Both arrows and pointed hands serve as adaptors and change perception of the pointing direction of test hands. However, the strength of the adaptation attenuated a little when participants adapted to arrows, showing a small yet significant difference between same and cross category adaptation. The finding that adaptation varies according to the type of adaptor confirms that pointed hands are a more effective adaptor than arrows, as evidenced by generally greater effect sizes.

The cross category adaptation observed suggests that a relatively simple orientation cue is driving the aftereffects here. The inclusion of a referent object to the scene increases the number of potential cues that participants could use in judging the pointing direction of the hand. These include the orientation of the index finger, the overall shape of the hand and forearm and additionally, vernier acuity—the misalignment of the tip of the skillet handle and the fingertip or tip of the arrow. In the case of vernier acuity this misalignment is identical for leftward pointing left hands and leftward pointing right hands but participants are more accurate in the latter than former case, thus suggesting that vernier acuity is not the dominant cue for the adaptation aftereffect.

When executing index finger pointing gestures a greater tendency to move the dominant right hand to extend further into contralateral space than the non-dominant left hand towards the right side of space has been reported [6]. Similarly, when observing pointing gestures participants respond faster to hand pointing stimuli that match their own dominant hand [32]. Similarly, where pointing and other gestures have been used in the hand laterality task, participants are faster to identify hands seen in egocentric than in allocentric perspective and reaction times are also modulated by participants’ own handedness [41,42]. In contrast, attention orienting to pointed hands has been shown to be independent of visual perspective, where response latency to same dominant hand pointing stimuli was not moderated when the viewpoint was switched from egocentric to allocentric visual perspectives [32]. While the participants in the present study did not produce the pointing gesture, the stimuli were shown from an egocentric, first person visual perspective.

While the dominant hand preference is pertinent in the context of observation of hand pointing it does not fully explain the finding of the present study that participants were more accurate at judging the direction of hands pointed toward the contralateral side of space than to those pointed towards the ipsilateral side of space. Further experimentation is needed to determine the exact cues that observers are using, and if superior accuracy for contralateral directed hand pointing stimuli is found in left-handers. However, our results show that we are incredibly accurate and can resolve pointing direction at very small angles and this fine-tuned discrimination is likely gauged from both the structure of the pointed hand and the extent that the stimulus extends diagonally into contralateral space.

Our findings are in line with a study [30] that found fine tuned visual discrimination of the morphological structure of the pointed index finger. Ariga and Watanabe [30] showed that hands pointing with the index finger are superior at directing attention than hands pointing with the middle or little finger. This advantage in manipulating the viewer’s direction of attention was shown not to be limited to the position of the index finger relative to the thumb or to the length of the index finger but from a holistic visual analysis of the morphological structure of the indexical gesture as a whole. Furthermore, when the accuracy and speed of directional judgments of index, middle and little finger at different lengths were measured, participants were fastest and most accurate at discriminating shortened index finger pointing stimuli. Interestingly, the type of indexical point did not always correlate with discrimination of direction, highlighting a partial dissociation between the cues we use to orient our attention to index finger pointing and those that we use to discriminate the direction of indexical pointing. However, both the present study’s finding of cross category adaptation to arrow direction and those of the study described above provide evidence that it is likely low level cues that drive both the attentional shift and direction discrimination.

A parallel can be drawn with the cues used to discriminate eye gaze direction. Studies of eye gaze perception have emphasized the importance of processing simple geometric cues such as scleral contrast, the relative position of the iris to the white matter of the eye [43]. Other studies have demonstrated the high resolution of gaze acuity [44–46]. Cline [45] reported very fine-tuned sensitivity to detecting small changes in eye gaze direction, as small as 1.4° from as far as 1 metre away. Given the importance of discerning eye gaze and hand pointing direction to social interaction, and the fact that gaze and pointing play similar roles in the development of shared attention and language acquisition, it is perhaps unsurprising that our perception of these cues is subserved by simple cues. However, it is important to note that in the case of eye gaze perception, using simple geometric cues alone only works under restricted settings as head orientation also contributes to the detection of eye gaze direction [47]. Similarly, the position of the index finger relative to the hand and the position of the forearm relative to the hand and body are further configural cues that we use to judge the direction of the point. Like eye gaze direction, it is likely a combination of simple geometric cues and configural processing at work in judging pointing direction.

The findings of the present study are unlike previous research that reported no transfer of aftereffects across categories [19,48]. Fang and He [19] employed cross category adaptation to examine the face viewpoint aftereffect and found no cross adaptation between images of human heads and of other non-biological objects, concluding that neurons selective for viewpoint are also selective for particular object categories [23]. Similar to the present study the stimulus categories used were biological (faces) and non-biological (wire like objects and cars). But, unlike those results [23], we observed cross adaptation between a non-biological directional cue (arrows) and a biological directional cue (pointed hands) presented oriented at or away from an object. Cross category orientation adaptation aftereffects between biological stimuli, heads and bodies, have been reported, where adapting to head orientation produces a perceptual bias in judging the turning direction of subsequently presented bodies. In contrast, little to no change in the judgment of head orientation occurs after adapting to extremely oriented bodies [49]. Similar cross category aftereffects have been reported along the higher order dimension of gender and shown to be independent of stimulus orientation [25]. In the case of the cross adaptation aftereffects reported here, cross-category adaptation to these two directional cues likely occurs at an early stage of visual processing.

Our finding that adapting to an arrow causes a shift in the perception of pointed hands suggests that it may not be the biological dimension of the stimuli per se that is producing the effects. Through experience we learn that both biological and non- biological cues contain salient directional information, so that our response to these cues is based on the directional information and not the semantic features of the stimulus. However, Bayliss et al [35] findings suggest otherwise, as adapting to a hand pointing stimulus did not result in weakened gaze cueing in the adapted direction while adapting to averted gaze did, which implies that it is the perception of the specific biological stimulus dimensions that modulates attention cueing. A strict comparison between studies that employed attention orienting and the present study’s use of perceptual adaptation is difficult, however by combining adaptation with subsequent measurements of attentional cueing, Bayliss et al [35] demonstrate a direct link between perceptual cues and attentional shifts.

To date very different research paradigms have been employed to examine the interaction of cues to social attention. Studies that employed attention cueing paradigms have shown that pointing elicits a spatial cueing effect in the same way as arrows and other directional cues. For example, Langton and Bruce [27] examined the relationship between the information present in the social directional cues; pointing gestures, head-gaze orientation and spoken directional information. Using a cross-modal interference paradigm, where the pointing gesture was either congruent or incongruent with the verbal stimulus “up” or “down” and the head orientation was congruent or incongruent with the pointing gesture, they observed bi-directional interference effects. Interestingly, in a control experiment they examined whether interference effects would be observed between head direction and non-social directional cues. The pointing stimuli were replaced with non-biological directional cues; arrows and participants were required to respond to the arrow and, in separate blocks, to the orientation of the head. The to-be-ignored head orientation cues were found to interfere with judgments of the direction of the arrow stimuli. This provides evidence that it is the directional meaning rather than the biological dimension of the stimulus that is encoded at this early stage of processing. Using a very different paradigm, our finding of strong cross-category adaptation is consistent with this interpretation.

Some neuroimaging studies point to a common neural mechanism for processing both biological and non-biological directional cues. An fMRI study that compared responses to passively viewed directional biological and non-biological stimuli showed similar response in the occipital temporal cortex to arrow and hand pointing stimuli [34]. Consistent with [28] where participants were required to actively follow either hand pointing or eye gaze stimuli, there was no differentiation in the response of STS to these stimuli, supporting the suggestion that STS could be involved in automatic attentional orienting towards the cued direction, regardless of the biological dimension of the stimulus.

The current study provides novel evidence of adaptation to hand pointing direction revealing separate mechanisms for coding right and leftward pointing directions. The precise stimulus features driving adaptation is still an open question as the cross adaptation effects suggest that adaptation may not be to the biological dimension of the stimulus but driven instead by simple orientation cues contained in the morphological structure of the pointed hand.
